# Abiotic drivers of activity in a large, free-ranging, freshwater teleost, Murray cod (*Maccullochella peelii*)

**DOI:** 10.1371/journal.pone.0198972

**Published:** 2018-06-08

**Authors:** Jason D. Thiem, Ian J. Wooden, Lee J. Baumgartner, Gavin L. Butler, Jamin Forbes, Matthew D. Taylor, Robyn J. Watts

**Affiliations:** 1 Department of Primary Industries, Narrandera Fisheries Centre, Narrandera, New South Wales, Australia; 2 Institute for Land, Water and Society, Charles Sturt University, Albury, New South Wales, Australia; 3 Department of Primary Industries, Grafton Fisheries Centre, Grafton, New South Wales, Australia; 4 Department of Primary Industries, Port Stephens Fisheries Institute, Taylors Beach, New South Wales, Australia; University of Tasmania, AUSTRALIA

## Abstract

The allocation of time and energy to different behaviours can impact survival and fitness, and ultimately influence population dynamics. Intrinsically, the rate at which animals expend energy is a key component in understanding how they interact with surrounding environments. Activity, derived through locomotion and basic metabolism, represents the principal energy cost for most animals, although it is rarely quantified in the field. We examined some abiotic drivers of variability in locomotor activity of a free-ranging freshwater predatory fish, Murray cod (*Maccullochella peelii*), for six months using tri-axial accelerometers. Murray cod (*n* = 20) occupied discrete river reaches and generally exhibited small-scale movements (<5 km). Activity was highest during crepuscular and nocturnal periods when water temperatures were warmest (19–30°C; January–March). As water temperatures cooled (9–21°C; April–June) Murray cod were active throughout the full diel cycle and dormant periods were rarely observed. Light level, water temperature and river discharge all had a significant, non-linear effect on activity. Activity peaked during low light levels, at water temperatures of ~20°C, and at discharge rates of ~400 ML d^-1^. The temporal changes observed in the behaviour of Murray cod likely reflect the complex interactions between physiological requirements and prey resource behaviour and availability in driving activity, and highlight the importance of empirical field data to inform bioenergetics models.

## Introduction

The time animals allocate to different behaviours has evolved around the principle of maximising energy gain whilst minimising predation risk [1]. Behaviours are rhythmic, typically consistent within species, are determined by a combination of biotic and abiotic stimuli, and are expressed as a trait that enables the optimal exploitation of a particular resource [2]. Plasticity in activity is a key behavioural trait that enables individuals to regulate responses to stochastic changes as well as predictable seasonal progression in abiotic and biotic stimuli [3].

Light levels and water temperature are predictable environmental factors that can influence diel and seasonal activity cycles (respectively) in fish. Diel variability in solar illumination results in predictable activity rhythms that centre on feeding and refuge seeking behaviour, and enables temporal partitioning of resources [2]. As most fish are ectothermic, changes in temperature ultimately control the rate of metabolic processes [4], and subsequently the capacity for locomotor activity [5]. But predictable rhythms often change in response to numerous endogenous factors including periods of spawning or parental care [3]. Temporary unpredictable changes, such as when an individual is exposed to increased predation pressure or a short-term fluctuation in environmental conditions, may also periodically alter activity rhythms [6]. However, there is also strong evidence of an evolutionary adaptation to circadian rhythms across many taxa [7], including aquatic organisms [8]. In lotic systems, variations in river flow, detected by fish as changes in fine-scale hydraulics, exert a strong influence on the availability of habitat, the supply of food resources and the energetic profitability of occupying a specific habitat. As such, river flow has a strong influence not simply on large-scale movements but also on fine-scale activity rhythms [9].

Murray cod (*Maccullochella peelii* Mitchell, 1839) is Australia’s largest freshwater fish, reaching a maximum size of 1.8 m in length and 113.5 kg in weight [10, 11]. Endemic to rivers of the Murray-Darling Basin, Murray cod occupy the top trophic position as a result of their size, gape and subsequently the ability to take large prey [12]. Little synchronicity has been observed in their seasonal movements suggesting individuals may respond in different ways to abiotic drivers [13]. In general, the species is considered site-attached and relatively sedentary throughout much of the year [13, 14]. No information documenting fine-scale movements or real-time activity patterns exist, although congeneric species have exhibited elevated activity during crepuscular (*M*. *mariensis* [15]) or nocturnal periods (*M*. *macquariensis* [16]; *M*. *ikei* [17]).

The aim of the current study was to quantify the relative importance of abiotic variables in determining the extent of Murray cod locomotor activity. The approach used in this study does not rely solely on an individual changing location *per se*, rather activity generated through body motion, including locomotor activity of site-attached individuals occupying discrete home ranges, is logged and transmitted. As Murray cod is considered largely sedentary based on observed daytime site-attachment [13, 18], methods that rely on an individual shifting location to generate movement indices may underestimate activity in this species. However, locomotor activity, which can incorporate both large and small spatial movements, can account for a large portion of an individual’s energy budget [19]. Incorporating field estimates of activity into bioenergetics models can represent a substantial deviation from simulated approaches [20], and is thus necessary for this and other species to improve model confidence and subsequently guide practical applications to fisheries management, such as river operations [21].

## Materials and methods

**Fig 1:**
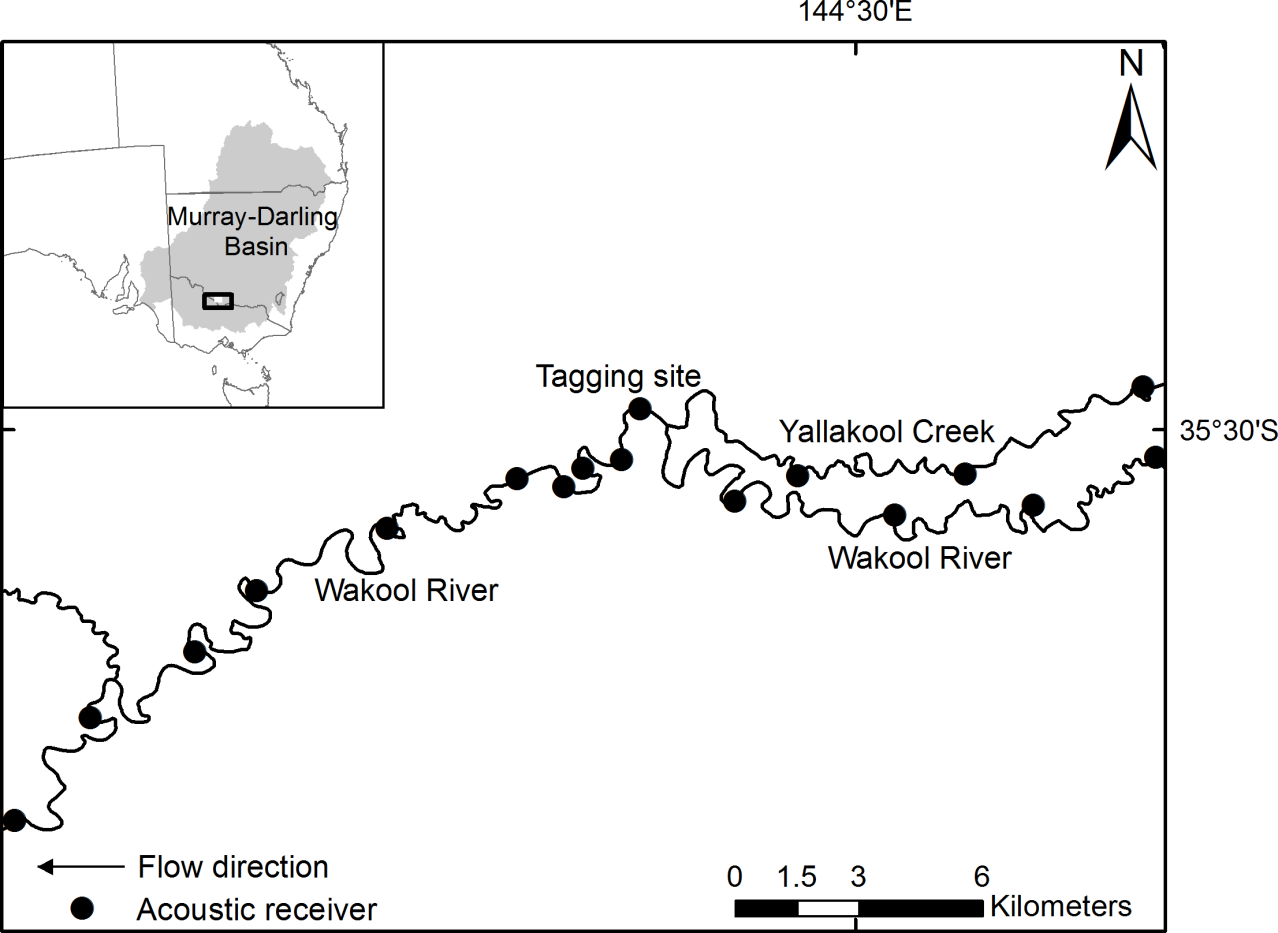
Location of the acoustic receiver array on the Wakool River and Yallakool Creek, south-eastern Australia, used to monitor locomotor activity of acoustically tagged Murray cod (*Maccullochella peelii*).

This study was undertaken in the Edward-Wakool river system, located in the southern Murray-Darling Basin in New South Wales, Australia. The Edward-Wakool is a large anabranch system of the Murray River, comprising over 1500 km of permanent and ephemeral rivers, creeks and flood runners, and wetlands [22] (Fig 1). The system is considered typical lowland river habitat for Murray cod, comprising slow-flowing turbid waters with deep holes and extensive amounts of in-stream large wood habitat.

We studied a ~55 km reach of riverine habitat, whereby tagged Murray cod were resident for an extended period of time such that near-continuous localised activity could be correlated with abiotic explanatory variables. Acoustic receivers (VR2W; Vemco Halifax Nova Scotia) were deployed in the Wakool River and Yallakool Creek in August 2015 (Fig 1). Receivers were deployed at approximately 6 km spacing to enable calculation of linear movement metrics. To maximise detection events and sample size, an additional three acoustic receivers were deployed at 1.5 km spacing between two of the 6 km receivers once the collection and tagging site for Murray cod was established. Murray cod (*n* = 20; 558–838 mm total length, 2.27–9.07 kg) were captured using boat-electrofishing and surgically implanted with acoustic telemetry accelerometer tags (model V13A-1L, 69 kHz, 42 mm length, 12.2 g in air; Vemco Halifax Nova Scotia) in August 2015. Tagging procedures were identical to those described by Butler et al. [23]. Individually numbered external dart tags were also attached to each fish to enable external identification, and unique passive integrated transponder tags were also inserted into the coelomic cavity of all individuals. The duration of each procedure took 5–10 min, after which fish were placed in aerated recovery tanks until they regained equilibrium and were subsequently released 11–48 min after surgery. Tags were inserted so that the longest dimension of the transmitter was parallel to fish length within the body cavity. Acceleration was sampled on three axes (x, y and z planes equivalent to surge, heave and sway) at 5 Hz for 15 sec with a measurement range of ±29.4 m s^-2^. A single integrated measure of locomotor activity was calculated as the root mean square of sampled acceleration from all three axes and was transmitted on average every 150 sec, in the range of 0–3.4 m s^-2^. These transmissions were decoded when transmitters were within detection range (~500 m) of submerged receivers.

River discharge data were obtained from the NSW Government WaterInfo website for upstream gauge stations on Yallakool Creek (gauge 409020 52 km upstream from the tagging site; accessed 5 September 2017) and Wakool River (gauge 409019 68 km upstream from the tagging site; accessed 5 September 2017). Average hourly discharge was determined for the study site by summing discharge rates from upstream gauge stations, combining this with discharge data from the Wakool Escape (not reported on the Waterinfo website and obtained from the New South Wales State Water Corporation (now WaterNSW)), which was adjusted to account for travel time (4 days) and estimated losses (20%; V. Kelly, WaterNSW pers. comm.). The bankfull discharge for Yallakool Creek and Wakool River is 4000 and 3000 ML d^-1^, respectively. However, discharge was below 520 ML d^-1^ for the study period, which is within the normal operating range for this system for delivery of irrigation water to downstream users (no unregulated flow events occurred in the study period). Thus, travel time and water losses from the gauges to the study site are well understood for this range of discharge. Average hourly water temperature was obtained from the nearest temperature logging gauging station located on the Wakool River (gauge 409062 73 km downstream from the tagging site; accessed 23 May 2017). Photosynthetically active radiation (PAR) was logged every ten minutes at the tagging site (Odyssey PAR Logger, Dataflow Systems Ltd Christchurch New Zealand).

### Data handling and analysis

Detection data were downloaded quarterly from acoustic receivers using Vemco User Environment software, and stored in a custom Microsoft Access database. Data were queried to encompass January–June 2016 inclusive for two reasons. Firstly, the purpose of this study was to examine the role of abiotic drivers on activity of Murray cod. Previous studies on other species have identified that the relationship between activity rhythms in fish and the abiotic drivers of these rhythms can break down during times of spawning and parental care [3], thus periods of potential spawning and parental care (September–December) were excluded. Secondly, the timing coincided with an extended period of high site fidelity (i.e. limited large-scale movement) for Murray cod, subsequently maximising sample sizes and data collection. Individual fish movements were recreated over time to visualise the spatial extent of river occupied during the study period. To explore the relative relationships between abiotic variables (independent predictor variables; river discharge, water temperature and light level) and relative activity (dependent variable), a generalised additive mixed model (GAMM, Gaussian family) was fitted. The dependent variable was created by calculating hourly medians of converted acceleration vectors, and centring these variables within each individual fish [24]. Independent variables similarly reflected hourly values. The model took the form *Act_ij_* = *β*_1_ + *s_ij_*(*PAR_j_*) + *s_ij_*(*Temp_j_*) + *s_ij_*(*Dsch_j_*) + *a_i_* + *ε_ij_* where *Act*_*ij*_ (activity) was the dependent variable for animal *i* at time point *j*, *Temp*_*j*_ was the temperature (°C), *PAR*_*j*_ was a relative measure of light level (unitless), *Dsch*_*j*_ was the river discharge rate (ML d^-1^), *a*_*i*_ was a random effect associated with individual animal *i* [25], *s* was a smoothing function, *β* was a constant, and ε was the residual error. Statistical analyses were conducted in R v. 3.2.0 [26] and used the mgcv package [27].

### Ethics statement

This research was carried out under Fisheries NSW Animal Care and Ethics permit 14/10 and Scientific Collection Permit P01/0059(A)-2.0.

## Results

### General findings

**Fig 2:**
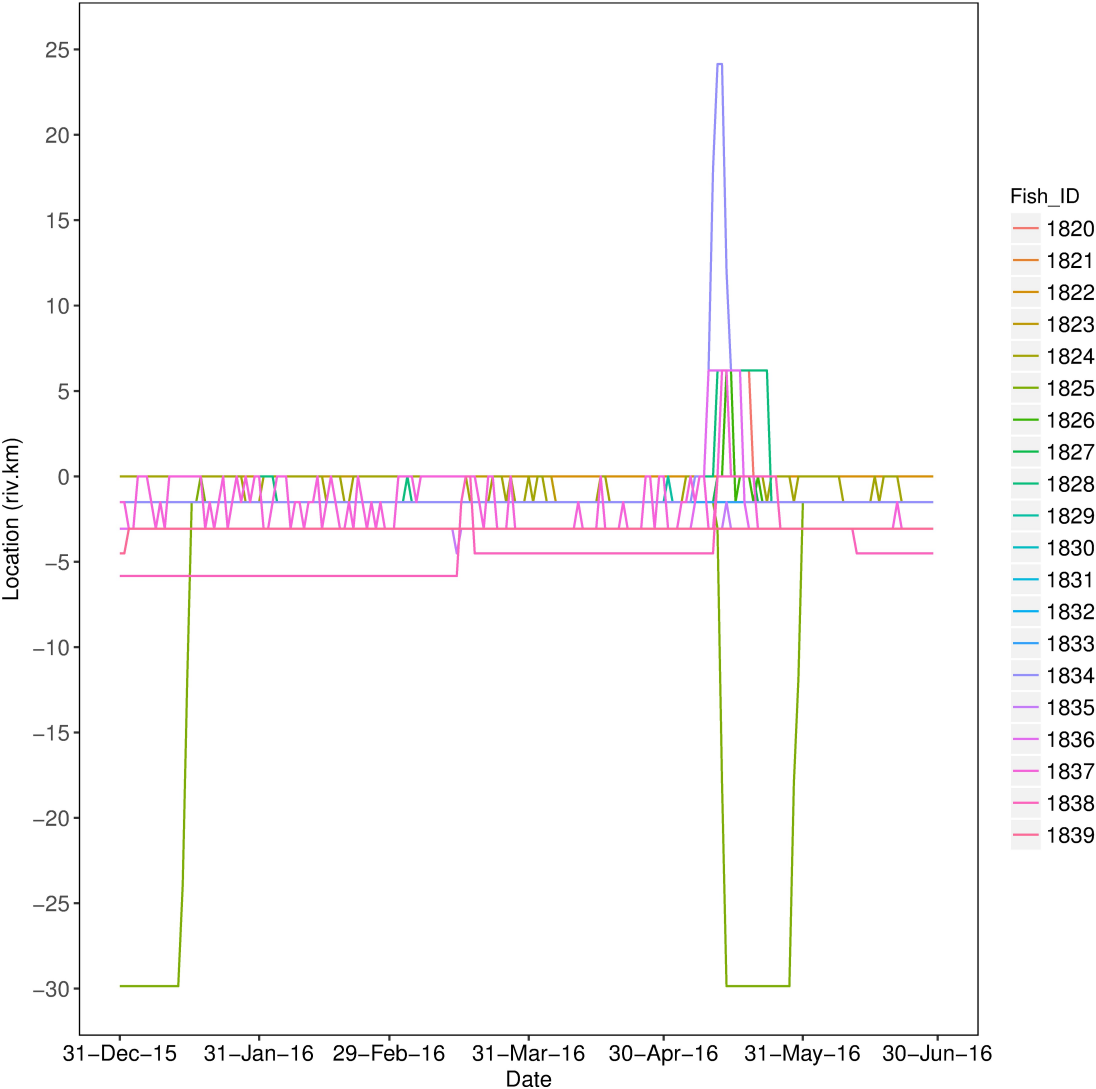
Daily location estimates of acoustically tagged Murray cod (*Maccullochella peelii*). Different coloured lines represent different tagged individuals and 0 km represents the tagging and release location of all individuals, with positive numbers representing upstream locations from the tagging site and negative numbers downstream locations.

**Fig 3:**
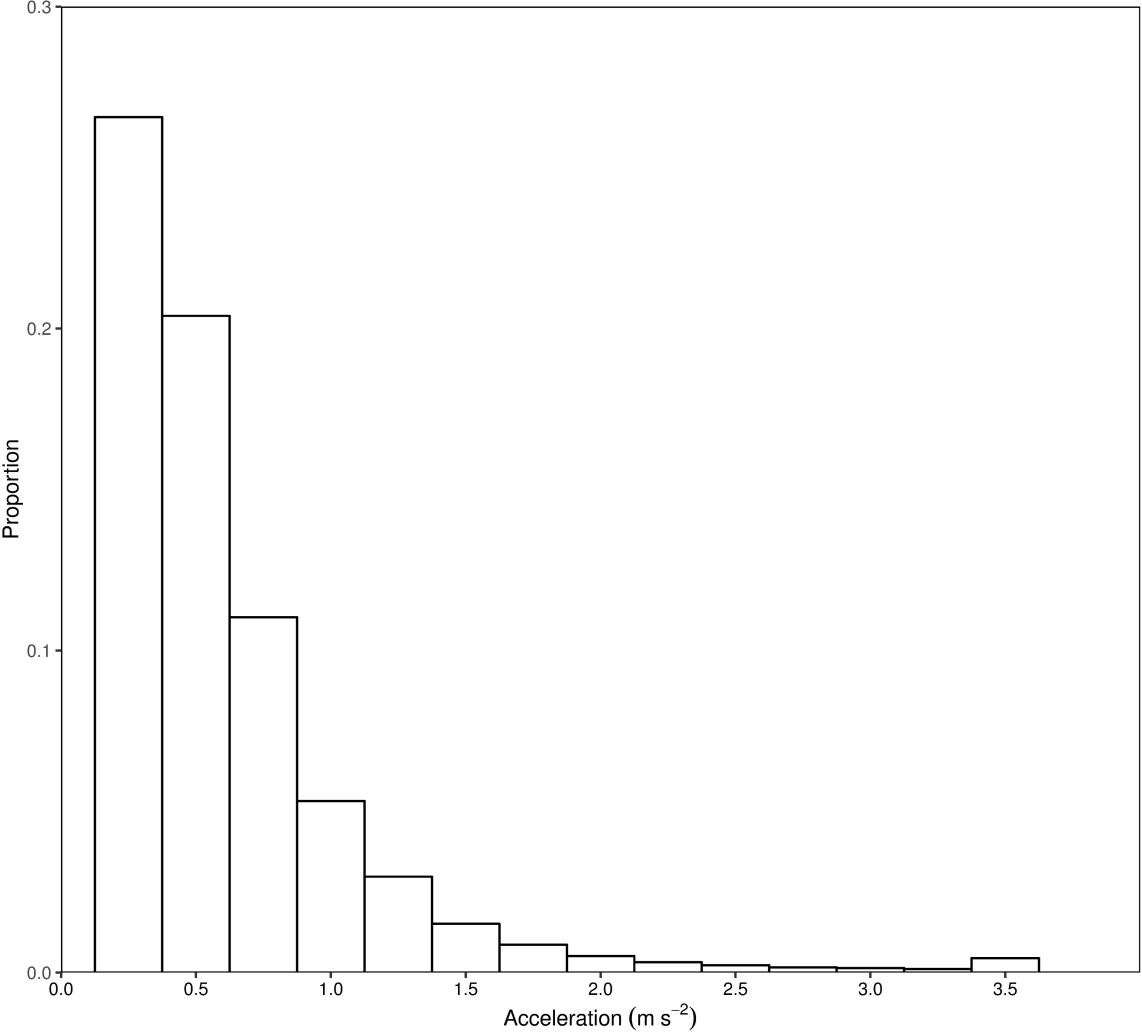
Frequency histogram of Murray cod (*Maccullochella peelii*) acceleration values measured during the study.

All 20 tagged Murray cod resided within the receiver array for the duration of the study (Fig 2). Murray cod movements spanned ~50 km of the Wakool River and Yallakool Creek, including ~30 km downstream and ~24 km upstream of the tagging and release site. Murray cod were predominantly located within a discrete reach of river at, or immediately downstream of, the tagging site. An exception occurred over a 2 week period from mid to late May where a number of individuals undertook rapid, temporary movements to upstream and downstream habitats (Fig 2). A total of 336,782 acceleration values were logged for the duration of the 6 month study from 18 individuals (Table 1). Two individuals were present in the study reach although did not contribute data as they resided between receivers (Table 1). The number of days Murray cod were detected ranged from 0–183 days, and 12 individuals were detected on more than 100 separate days (Table 1). Acceleration values ranged between 0 and 3.46 m s^-2^, although the majority of values were below 1 m s^-2^ (Fig 3).

**Table 1:** Summary information on individual Murray cod (*Maccullochella peelii*) fitted with accelerometer acoustic telemetry tags and monitored from January–June 2016.

Fish ID	Total length (mm)	Weight (g)	Sex	Detections	Days detected
1820	748	6182	Female	54393	178
1821	625	3026	Female	2821	161
1822	724	6126	Unknown	4142	152
1823	651	3680	Unknown	154	2
1824	796	7452	Female	7821	131
1825	678	3878	Female	4025	127
1826	670	3170	Female	44746	171
1827	618	3120	Unknown	12796	176
1828	645	3234	Unknown	6328	153
1829	598	2576	Male	59305	183
1830	605	2898	Female	0	0
1831	558	2274	Male	30	5
1832	693	4896	Female	604	20
1833	638	3666	Unknown	0	0
1834	628	3376	Unknown	316	12
1835	838	9070	Female	7468	143
1836	628	3090	Unknown	75213	176
1837	689	5064	Unknown	56374	166
1838	665	3626	Unknown	217	7
1839	593	2656	Male	29	2

Water temperature ranged from 9–30°C during the study, transitioning from warm temperatures in Austral summer (January 2016) to cooler temperatures in Austral winter (June 2016) and were largely reflective of the annual extremes typically encountered by Murray cod in this region. PAR values were characterised by regular daily cycles and seasonal decline. River discharge ranged between 200 and 400 ML d^-1^ from January to May, peaked at 518 ML d^-1^ on 8 May 2016 after which it steadily declined to 0 ML d^-1^ on 21 May 2016 and remained at this rate until the cessation of the study.

### Abiotic drivers of activity

**Fig 4:**
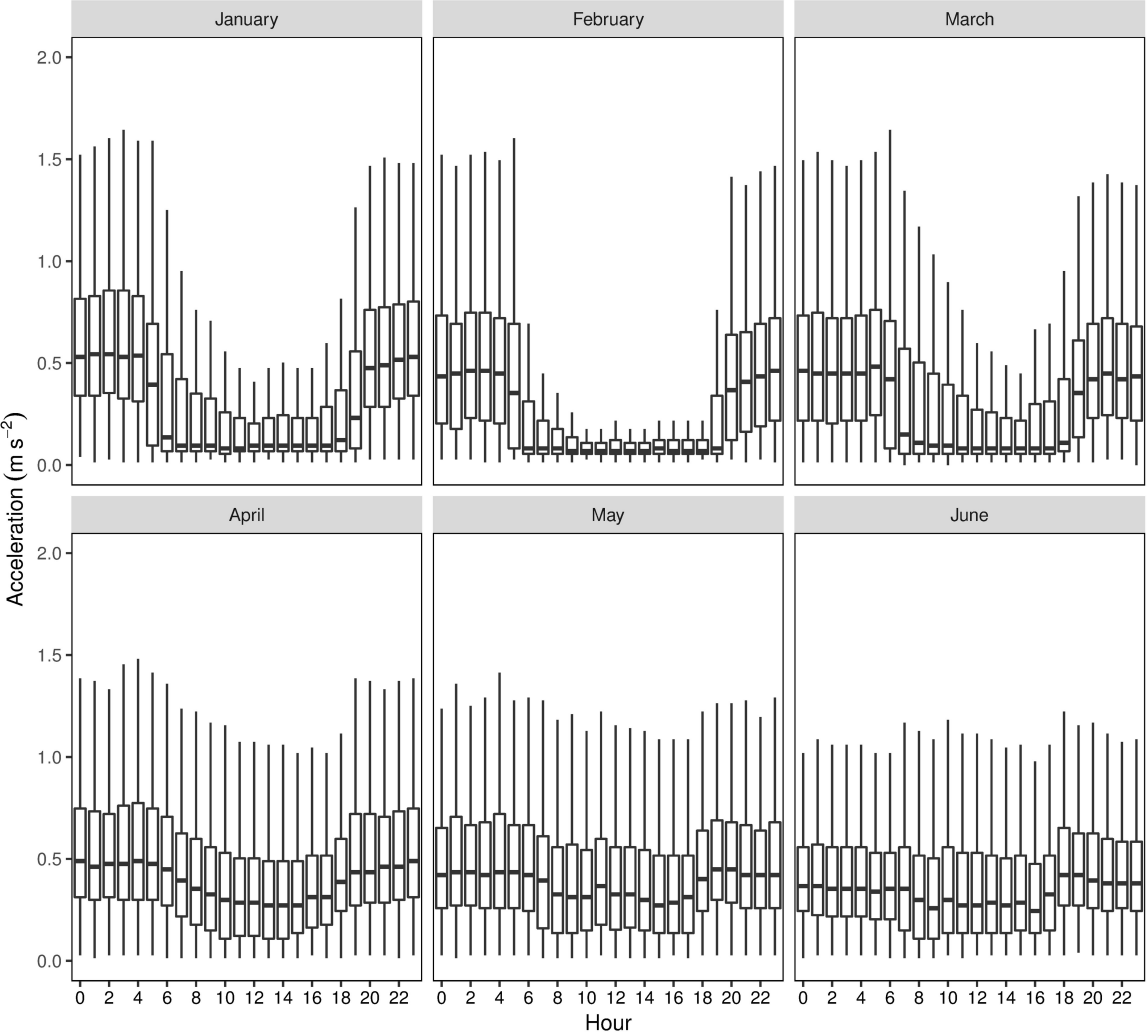
Combined hourly acceleration values of Murray cod (*Maccullochella peelii*; n = 18) for each calendar month of the study. Data are represented as median, 25^th^ and 75^th^ percentiles (box) and 5^th^ and 95^th^ percentiles (whisker).

**Fig 5:**
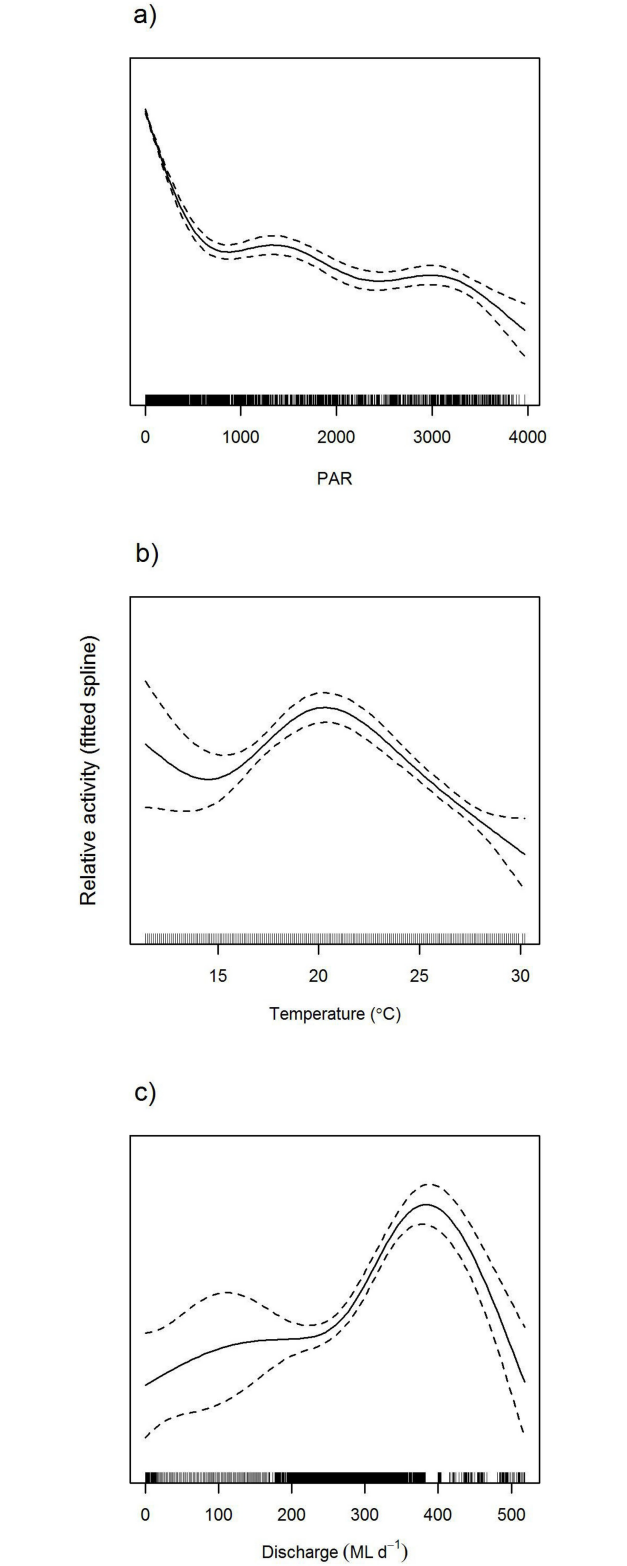
GAMM smoothing curve indicating relative activity patterns of Murray cod (*Maccullochella peelii*) (on *y*-axis, note that these are a unitless, relative measure of the dependent variable) for partial effects of photosynthetically active radiation (a.; PAR, a continuous variable reflecting light intensity), water temperature (b.), and river discharge (c.). Dashed lines are 95% confidence intervals.

Murray cod exhibited distinct patterns in locomotor activity, with activity appearing to peak during crepuscular and nocturnal periods, particularly during warmer water temperatures (Fig 4). Modelling identified a significant relationship between PAR and activity (e.d.f. = 4.09, *F*_4.9_ = 552.65, P << 0.001; Fig 5A). Activity decreased over the entire range of PAR values measured, but the decrease was much sharper at lower ambient light levels. These patterns suggest a substantial modulating effect of light on activity levels. Similarly, temperature had a significant non-linear relationship with activity (e.d.f. = 4.51, *F*_4.5_ = 35.25, P << 0.001; Fig 5B). Activity was highest at intermediate temperatures (~20°C), with lower activity either side of this peak. River discharge also had a significant effect on activity (e.d.f. = 4.60, *F*_4.6_ = 32.84, P << 0.001; Fig 5C). There was only a minor effect on activity at lower rates of discharge; however, when discharge levels exceeded ~200 ML d^-1^, activity increased sharply up to discharge rates of ~400 ML d^-1^. Activity rapidly decreased for discharge rates between 400 ML d^-1^, and the maximum discharge rates observed (~520 ML d^-1^).

## Discussion

For the duration of this study, Murray cod exhibited strong patterns in locomotor activity whilst occupying relatively discrete river reaches. Previous studies have revealed that seasonal longitudinal riverine movements of Murray cod generally peak in the Austral spring (September–November), coinciding with the species breeding period, although the timing and magnitude of movements varies among individuals and between years [13, 14]. As such, while this current study was theoretically undertaken within a period of limited movement, activity values reflect patterns of localised movement by Murray cod. Light level, water temperature and river discharge all exerted a significant effect on the activity of Murray cod. Distinct nocturnal activity patterns were particularly evident during warmer water temperatures (January–March) which are consistent with studies of congeneric trout cod (*M*. *macquariensis* [16]) and eastern freshwater cod (*M*. *ikei* [17]). As water temperatures cooled (April–June), Murray cod displayed elevated diurnal activity (with little change in nocturnal activity). This temporal change resulted in continuous activity throughout the entire diel period during this time. There is no data to support comparisons of this behaviour with congeneric species, although Thiem et al. [28] previously identified increased winter diurnal activity of confamilial Macquarie perch (*Macquaria australasica*).

The measurements of field locomotor activity recorded for Murray cod in this study are in units of dynamic acceleration, which correlate directly to the swimming speed of fish (e.g. [29]) and subsequently represent their rate of work or energy expenditure [30]. As most fish are ectotherms, water temperature has a large effect on metabolic processes, thus determining the energy available for activities such as locomotion [4]. For most fish species, this control is manifested through an optimal swimming performance curve in relation to water temperature [5]. Murray cod exhibited increased activity up to ~20°C which declined thereafter. Whiterod [31], however, identified that juvenile Murray cod generally lacked greater performance at specific temperatures, suggesting potential adaptation to thermal fluctuations in their environment. The cause of the discrepancy between the two studies is unknown, although likely relates to differences in the methodology used to assess performance, as well as ontogeny.

River discharge had a significant effect on the activity of Murray cod, with activity peaking at discharge rates of ~400 ML d^-1^ and declining up until 520 ML d^-1^ (the maximum discharge experienced in this study). In a meta-analyses of fish movement, Taylor and Cooke [9] identified that during non-migratory periods fish are increasingly more likely to shift location as discharge rates increase, but not necessarily increase locomotor activity which does not exclusively require a change in location. This latter relationship may be explained by local-scale hydraulic diversity, functional morphology and/or behavioural responses. For example, Taylor et al. [32] found that during high flow releases from a hydropeaking dam, bull trout (*Salvelinus confluentus*) often reduced activity, and thus conserved energy, through either flow refuging (i.e. occupying low flow habitats created by instream habitat) or station holding (resting on the bottom against hard substrate). This explanation appears unlikely for Murray cod in the current study, as when discharge rates are between 170 and 800 ML d^-1^ the majority of the study reach has velocities ranging between 0.02–0.30 m s^-1^ [33]. However, further investigation across a wider range of discharge values is required to confirm these patterns, as modelling indicates it could have a substantial effect on energetics [34]. In the current study, it is more likely that a temporary shift in river discharge resulted in Murray cod altering a predictable activity rhythm. Payne et al. [6] previously identified rainfall-induced reversal in the activity rhythms of yellowfin bream (*Acanthopagrus australis*), and attributed the change in behaviour to a need to forage more often due to reduced daytime feeding efficiency at high turbidity levels. While the mechanism contributing to increased activity of Murray cod at specific river discharge rates remain unknown, feeding efficiency or temporary prey resource exploitation represent a plausible explanation.

Murray cod are considered an apex predator due to their ability to feed on other macrophagic carnivores [12]. Despite this classification, decapods such as freshwater prawns (*Macrobrachium australiense*) and common yabby (*Cherax destructor*) are numerically the most abundant prey items consumed by Murray cod [12, 35]. The former are seasonally more abundant at warmer water temperatures [36] and can form large aggregations during summer and early autumn (e.g. [37]). Moreover, freshwater prawns exhibit strongly nocturnal activity, often sheltering in dense cover during the day and foraging in the open at night [38, 39]. The availability and behaviour of this prey resource during warmer water temperatures may therefore explain the consistent nocturnal activity of Murray cod from January–March.

Temporal variation in Murray cod diet is unknown, although the closely related *M*. *ikei* display dietary plasticity across seasons and an ontogenetic shift towards larger prey items as they grow [40]. A seasonal shift in prey type represents another possible explanation for the resultant progressive change in diel behaviour of Murray cod. Shifts in prey type and availability have been extensively documented to alter diel activity rhythms in other species. For example, both brown trout (*Salmo trutta*) and rainbow trout (*Oncorhynchus mykiss*) alter diel activity to opportunistically exploit changing food resources [41]. While metabolic processes may be slower at cooler water temperatures, thus reducing the required energy intake for growth, patchily distributed prey resources or an alteration in prey behaviour may account for the temporal shift in Murray cod locomotor activity. Importantly, although metabolic processes are reduced in winter, Murray cod divert energy resources into gonad development from as early as April even though spawning does not occur until September through to November, thus highlighting the significance of diet and condition over winter [42].

## Conclusions

In the current study we measured locomotor activity of a large freshwater teleost, Murray cod, and determined that light availability, water temperature and river discharge all influence field activity. We also propose that unquantified biotic factors including seasonal prey availability and behaviour may be influencing Murray cod activity. In combination, these findings are not only important for understanding the biology of the species but also have implications for the long-term management of the species. Altered river flows and water temperatures resulting from river regulation have contributed to declines in Murray cod populations [11]. High water velocities and depressed water temperatures immediately downstream from large dams also inhibit the growth of Murray cod [21]. Murray cod in the current study altered activity in response to changes in river discharge and water temperature, confirming that both parameters directly influence the behaviour of the species. Field measurements of activity in fish are rarely obtained, although can account for a highly variable proportion of the active metabolic rate, subsequently affecting consumption estimates and bioenergetics budgets [19, 20]. Following calibration of accelerometer output with oxygen consumption in a swim-tunnel respirometer (e.g. [29]), the results from this study can be used to refine the activity component of existing bioenergetics models for the species [34]. This would improve use of management practices such as dam operation (e.g. controlled flow and temperature regimes) to enhance population outcomes for the species [21].

## Supporting information

S1 File Complete dataset used in analysis, including explanation of data. (XLSX)
